# Comparative Analysis of the Histological Characteristics of Bone Tissue Following Implant Drill Preparation Under Various Parameters: An In Vitro Study

**DOI:** 10.3390/jcm14072161

**Published:** 2025-03-21

**Authors:** Piotr Kosior, Maciej Dobrzyński, Kamila Wiśniewska, Michał Kulus, Natalia Struzik, Jacek Matys, Piotr Kuropka

**Affiliations:** 1 Department of Conservative Dentistry with Endodontics, Wroclaw Medical University, Krakowska 26, 50-425 Wroclaw, Poland; 2 Department of Pediatric Dentistry and Preclinical Dentistry, Wroclaw Medical University, Krakowska 26, 50-425 Wroclaw, Poland; maciej.dobrzynski@umw.edu.pl; 3 Dental Surgery Department, Wroclaw Medical University, Krakowska 26, 50-425 Wroclaw, Poland; kamila.wisniewska@umw.edu.pl; 4 Division of Ultrastructural Research, Wroclaw Medical University, Chałubińskiego 6a, 50-368 Wrocław, Poland; michal.kulus@umw.edu.pl; 5 Pre-Clinical Research Centre, Wroclaw Medical University, Bujwida 44, 50-368 Wroclaw, Poland; 6 Department of Histology and Embryology, Wroclaw University of Environmental and Life Sciences, Norwida 25, 50-375 Wrocław, Poland; piotr.kuropka@upwr.edu.pl

**Keywords:** implantological drills, osseointegration, thermal bone damage

## Abstract

**Purpose:** This study aimed to compare the histological characteristics of bone tissue following drilling with three implant systems under different rotational speeds and cooling conditions. **Methods:** A total of 54 implant bed preparations were performed in four swine ribs using three implant systems: Hiossen ET (Hiossen, Fairfield, NJ, USA), Paltop (Burlington, MA, USA), and Anyridge (Megagen, Daegu, Republic of Korea). Drilling was performed at three speeds (800, 1200, and 1500 rpm) under three cooling conditions: saline at room temperature, saline cooled to 4 °C, and no cooling. Histological evaluation was conducted using a Nikon Eclipse 80i fluorescence microscope (Nikon, Tokyo, Japan) with DAPI and rhodamine staining. Observations were performed at 40× magnification, focusing on the osteotomy wall and surrounding tissue. The samples were assessed based on surface smoothness, compressed tissue presence, carbonization, and adjacent tissue damage. Statistical analysis was performed using the Kruskal-Wallis test with Dunn’s post hoc comparisons to evaluate differences among experimental conditions. **Results:** The results demonstrated that the Hiossen ET system achieved optimal bone bed quality at 1200 rpm with saline cooling at 4 °C, producing the smoothest osteotomy walls and minimal thermal damage (*p* = 0.003). The Paltop system performed best at 800 rpm with 4 °C cooling, showing reduced tissue compression and fewer microcracks (*p* = 0.012). The Anyridge system exhibited the most favorable outcomes at 1200 rpm with saline cooling at room temperature, minimizing soft tissue remnants and preserving bone integrity (*p* = 0.021). Across all systems, the absence of cooling significantly increased thermal damage, carbonization, and tissue fragmentation, particularly at 1500 rpm (*p* < 0.001). **Conclusions:** The use of lower rotational speeds with effective cooling minimized tissue trauma and improved bone bed integrity. Further clinical validation is necessary to confirm the applicability of these results in human bone.

## 1. Introduction

The success of implantology procedures relies heavily on the precise preparation of the bone bed, which serves as the foundation for achieving optimal primary stability and long-term osseointegration [1,2]. Current implant protocols encompass a variety of techniques, including machine-driven rotary systems, ultrasound devices, laser systems, and manual systems [3,4,5,6]. Among these, rotary systems dominate clinical practice due to their efficiency, predictability, and well-established protocols. These systems employ a sequential series of drills to create the desired osteotomy, facilitating the placement of the implant while preserving the structural integrity of the bone [7,8]. During the drilling process, bone tissue undergoes mechanical deformation, which may manifest as resilient or elastic changes [9]. The mechanical behavior of bone is influenced by its material properties, particularly Young’s modulus of elasticity, which reflects its capacity to withstand deformation under stress. This parameter is crucial in determining the biomechanical response of bone to drilling forces and its subsequent ability to support osseointegration. Despite the widespread use of rotary systems, all bone preparation techniques share a common objective: to minimize trauma to the bone tissue while preserving its biological viability and regenerative capacity [10,11,12].

A key factor influencing tissue viability is temperature. Studies have consistently shown that sustained temperatures above 47 °C for more than one minute can denature the bone’s organic matrix, leading to irreversible damage and necrosis [13,14,15]. Thermal damage during drilling is affected by several interrelated factors, including rotational speed, drill design and morphology, applied pressure, and the cooling technique employed. Effective cooling is particularly critical in mitigating heat generation and protecting the surrounding bone from thermal injury. Research has demonstrated that inadequate cooling or excessive rotational speeds can lead to significant temperature elevations, adversely impacting the structural and cellular integrity of bone tissue [16,17,18]. Furthermore, the morphology and surface finish of the drill play a role in reducing friction and heat generation, thereby contributing to the preservation of bone viability.

A wide range of drill systems is available on the market, each characterized by specific parameters such as design, length, and manufacturer-recommended operating guidelines. These variations significantly influence the preparation process, as inappropriate parameter settings can compromise the quality of the prepared bone bed due to the distinct characteristics of each system [19,20]. Among these parameters, rotational speed plays a critical role in ensuring effective and safe drilling. Maintaining an optimal rotational speed is essential to prevent excessive heat generation, which can adversely affect bone viability [21,22]. Failure to adhere to appropriate speed settings forces the operator to exert greater pressure on the drill, thereby increasing friction and raising the temperature within the drilling site [21]. Elevated rotational speeds can further exacerbate this issue, as the increased frequency of drill-to-bone contact generates additional heat. Effective cooling during the drilling process has been shown to mitigate thermal damage and improve the quality of the bone bed [23]. Saline solution, commonly used for cooling, should be maintained at or below room temperature to maximize its effectiveness [24].

The micromorphology of the prepared bone bed is critical for both initial stabilization and the long-term osseointegration process [25]. Successful osseointegration requires direct contact between the prepared bone surface and the implant [26]. Minimizing the production of loose, torn, or necrotic bone fragments during drilling is crucial, as these fragments can act as physical barriers that disrupt the contact between bone-forming cells and the implant surface. Furthermore, the presence of such debris may trigger an immune response, potentially impairing the healing process [11]. The quality of the bone surrounding the implant site is equally important. Cracks, microfractures, or other forms of damage in the vicinity of the implant bed can significantly hinder osseointegration. Such damage may compromise the stability of the implant and, in severe cases, lead to implant failure or rejection [27,28].

The objective of this study was to evaluate and compare the histological differences in bone tissue prepared with drill systems from three different manufacturers—Hiossen ET (Hiossen, NJ, USA), Anyridge (Megagen, Republic of Korea), and Paltop (Burlington, MA, USA)—under varying rotational speeds and cooling conditions. This analysis aims to highlight the impact of these parameters on bone bed quality and provide insights into optimizing implant preparation techniques. These findings enhance the understanding of how drilling parameters impact osseointegration and provide valuable clinical guidelines for optimizing implant success rates.

## 2. Materials and Methods

### 2.1. Sample Preparation

The present study utilized four right-side swine ribs, in which a total of 54 implant bed preparations (n = 54) were performed. The specimens were prepared using drills from three different implant systems, Hiossen ET (Hiossen, NJ, USA), Anyridge (Megagen, Republic of Korea), and Paltop (Burlington, MA, USA), at varying rotational speeds. Specifically, the drills were tested at three speeds (800, 1200, and 1500 rpm) under three cooling conditions: saline at room temperature, saline cooled to 4 °C, and no cooling.

Bone bed preparation followed a standardized sequential protocol, with drills used to create osteotomies for the smallest implant diameter recommended for each system. This approach ensured consistency across systems and allowed for a controlled comparison of the effects of rotational speed and cooling on bone quality.

**Figure 1:**
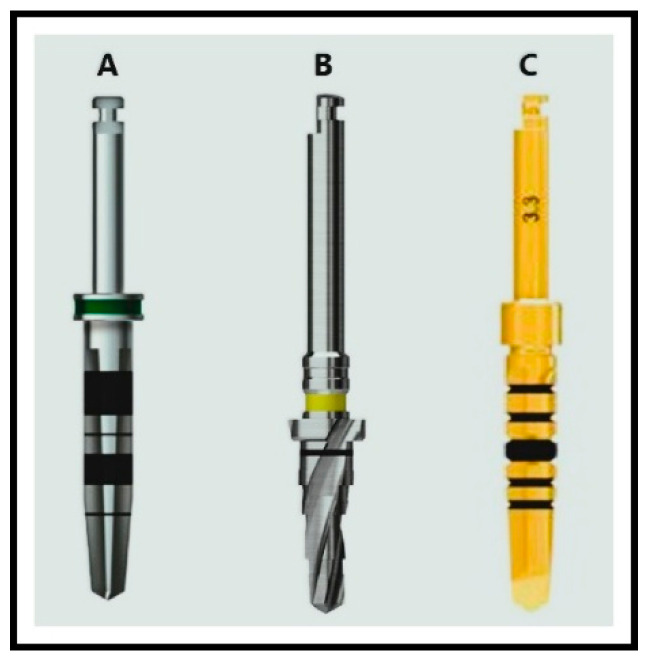
Drill systems used in the study: **A**: Paltop^®^ system, **B**: Hiossen^®^ system, **C**: AnyRidge^®^ system.

The drill diameters used for each system were as follows:

- Paltop: 2.0 mm, 2.4 mm, 3.75 mm
- Hiossen: 2.0 mm, 2.2 mm, 3.5 mm
- AnyRidge: 2.5 mm, 2.8 mm, 3.3 mm (Figure 1)

All implant beds were prepared to a depth of 10 mm and a width corresponding to the final drill in the sequence for each system.

The geometry of the drill bits was assessed using the the Discovery V20 stereo microscope (Carl Zeiss Microscopy GmbH, Jena, Germany).

For drilling, the NeoSurge implant engine (NEO BIOTECH, Wonjusi, Republic of Korea, Saeshin United, Seoul, Republic of Korea) was used with an auto-reverse option, equipped with a 32:1 contra-angle handpiece. Each hole was drilled three times. Firstly, an initial hole (with the smallest diameter) was created, which was then reamed twice to achieve the final diameter and shape of the osteotomy.

### 2.2. Study Groups

The study groups were organized based on the implant system used. Three groups were formed, each corresponding to one of the tested implant systems: Hiossen ET (Hiossen, NJ, USA), Anyridge (Megagen, Republic of Korea), and Paltop (Burlington, MA, USA). Each group consisted of 18 samples (implant bed preparations), resulting in a total of 54 samples across all groups.

Within each implant system group, samples were further divided based on the drilling speed. Implant beds were prepared at three rotational speeds: 800 rpm, 1200 rpm, and 1500 rpm, with six samples assigned to each speed.

Each speed condition was then subdivided based on the cooling method used during drilling. Three cooling conditions were applied:

- Saline at room temperaturę (n = 6)
- Saline cooled to 4 °C (n = 6)
- No cooling (n = 6)

For each implant system and each drilling speed, two samples were prepared under each cooling condition, ensuring an equal distribution of samples across all experimental conditions.

The implant bed preparations were performed following a previously published protocol, ensuring consistency and reproducibility of the drilling process [29].

### 2.3. Fluorescence Microscopy Analysis

The autofluorescence properties of the material were utilized for direct evaluation using a Nikon Eclipse 80i fluorescence microscope (Nikon, Tokyo, Japan). To visualize the presence of cells and non-mineralized components, the samples were stained with a combination of DAPI and rhodamine, enabling clear differentiation of the morphology of the well edges. Fluorescence excitation was performed using a UV-2A filter (excitation range 330–380 nm, barrier filter at 420 nm). Observations were conducted at 40× magnification, focusing on the region where the first complete circular structure was formed by the drill.

### 2.4. Qualitative Evaluation Criteria

A descriptive qualitative evaluation was conducted to assess the quality of the prepared implant beds. An experienced histologist with expertise in bone tissue assessment evaluated the implant bed quality qualitatively. The evaluator underwent a calibration process before the study to ensure consistency and reliability. This process involved repeated assessment of a subset of samples to verify intra-rater reliability. The following parameters and criteria were adopted:

Parameter 1: Surface Smoothness (Evaluated for torn holes, uneven edges, and debris)

Parameter 2: Compressed Tissue Presence (Assessed for compacted tissues)

Parameter 3: Carbonization (Evaluated for signs of tissue carbonization)

Parameter 4: Cracking and Adjacent Tissue Damage (Evaluated for damage to tissues surrounding the drill hole)

Each parameter is rated on a scale from 1 to 4, with 4 indicating no adverse change. Maximum total score per sample = 16 (sum of all parameters). Classification based on total score:

0–7 = Poor quality
8–11 = Medium quality
12–16 = Good quality (Table 1)

### 2.5. Statistical Analysis

The statistical methods used in the current study are focused on finding the variables that most significantly influence the quality of implant drilling and on finding the best combination of implantation system, rotation speed, and cooling conditions. Since the scores obtained in the study are non-parametric, nested ANOVA and other parametric tests could not be applied; a non-parametric Kruskal-Wallis test with Dunn’s post hoc comparisons was used.

Statistical analysis was supported with Jamovi (Version 2.3) [30] and R statistical environment [31].

## 3. Results

A comparative analysis of the rib bone structure was conducted using Hiossen, Anyridge, and Paltop drill bits at varying speeds with and without modified cooling.

### 3.1. Hiossen ET—Fluorescence Microscopy Analysis

**Figure 2:**
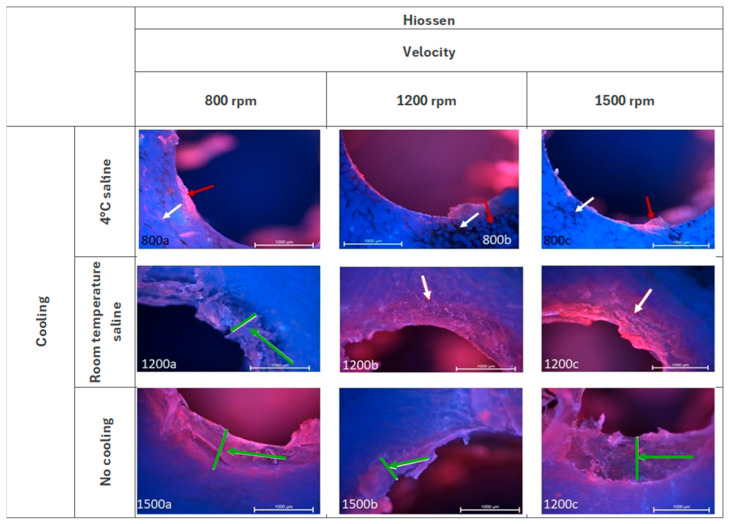
Microphotographs of the edges of the Hiossen group of holes. DAPI and Rhodamine. Magnification 40×.

As illustrated in Figure 2, the Hiossen group exhibits a remarkably uniform surface with cooling at temperature of 4 °C, particularly in the context of well wall characteristics. The adjacent tissues have undergone a process of cracking (white arrow). There is minimal deposition of soft tissue (red arrow) on the surface. When cooling at room temperature was implemented, residual soft tissues are observed in the form of a compression zone (green arrow) containing an irregular mixture of tissues. Evidence of adjacent tissue cracking is discernible at higher velocities (white arrow). When working without cooling, the presence of residual soft tissue is indicated by the observation of a compression zone (green arrow) comprising an irregular mixture of tissues. This phenomenon is further accentuated when higher velocities are employed. The adjacent tissues exhibited partial degradation at speeds of 1200 rpm and 1500 rpm.

### 3.2. Anyridge—Fluorescence Microscopy Analysis

**Figure 3:**
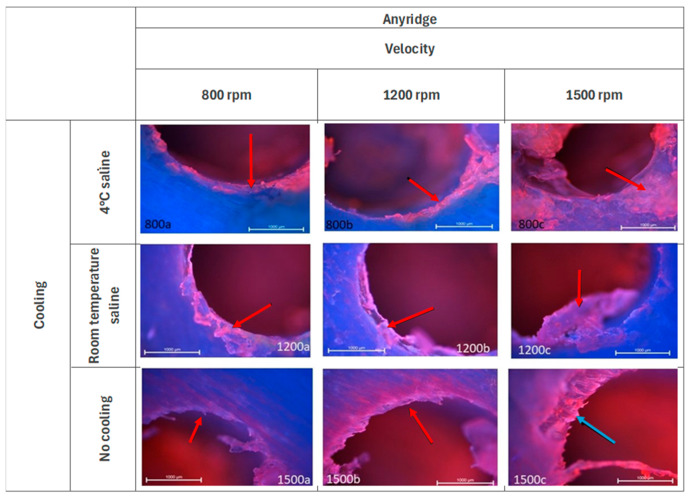
Microphotographs of the edges of the Anyridge group of holes. DAPI and Rhodamine. Magnification 40×.

As illustrated in Figure 3, with a cooling temperature of 4 °C, there are discernible jerking effects observable at speed 800 rpm. At speed 1200 rpm, there are visible “ground tissues” (red arrow), which consist of torn fibers and remnants of soft material. At speed 1500 rpm similar structures are observed (red arrow). When cooling at a room temperature, residual tissue elements adhering to the well wall are discernible at speeds 800 rpm and 1200 rpm. At speed 1500 rpm, the surface is observed to be smooth, yet larger tissue fragments are noted to be attached to the bone (red arrow). No damage was observed in the adjacent tissues. At speed 1500 rpm without cooling, the effects of drill jerking are evident (blue arrow). At all speeds, fibers associated with the borehole wall are discernible. The adjacent tissues remain unaltered.

### 3.3. Paltop—Fluorescence Microscopy Analysis

**Figure 4:**
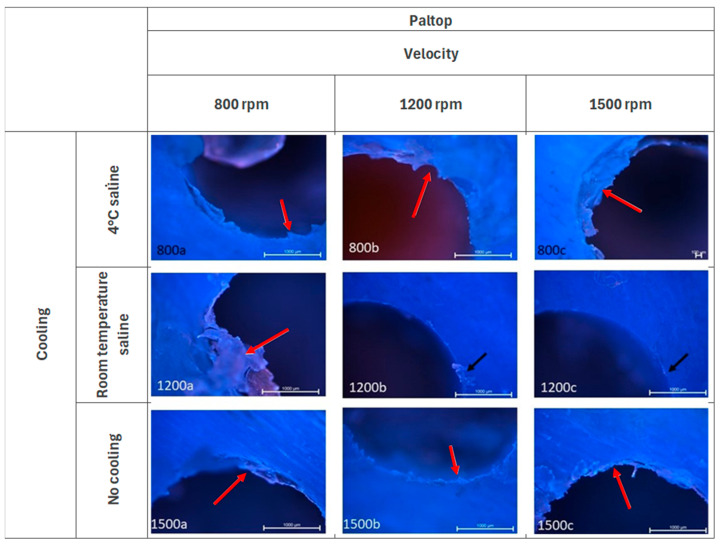
Microphotographs of the edges of the Paltop group of holes. DAPI and Rhodamine. Magnification 40×.

In the Paltop group (Figure 4), with a cooling temperature of 4 °C, evidence of torn tissue remains visible at speeds 800 rpm and 1200 rpm. At speed 1500 rpm, the surface is observed to be smooth, with adjacent tissues remaining undamaged. However, remnants are still evident in proximity to the well wall (red arrow). When cooling at room temperature, fine carbonization in the form of small fragments is visible at all speeds (black arrow). The greatest fragmentation is observed at velocity of 1500 rpm and the least at velocity of 800 rpm. When working without cooling, there are residues still associated with the well wall (red arrow). The surface damage is significantly more pronounced, with tissue fragments forming a ring around the borehole wall (red arrow) and varying degrees of damage.

### 3.4. Influence of Distinct Drilling Parameters on Implantation Quality

As for the implantation system, the Hiossen system gives significantly higher scores than both Anyridge and Paltop for Parameter 1 and higher than Anyridge for Parameter 2. The lowest speed (800 rpm) gives a better score than the highest in all parameter categories and better than 1200 rpm for Parameters 1 and 4.

**Figure 5:**
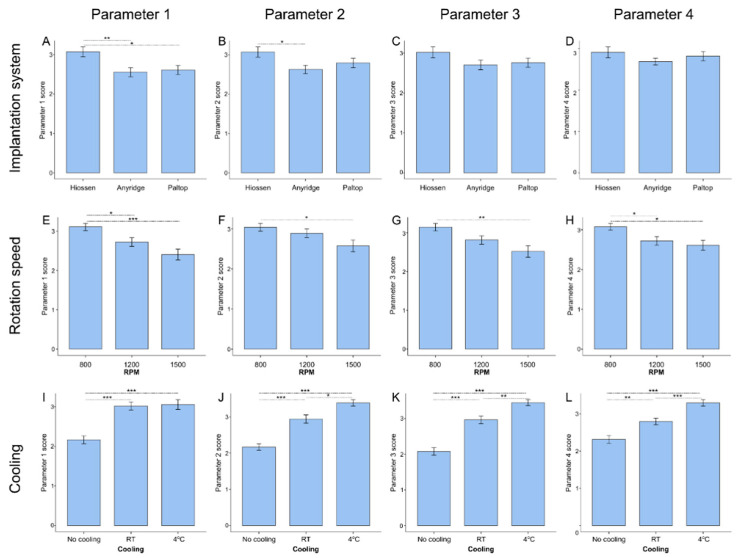
Comparison of different drilling parameters according to four evaluated categories. (**A**–**D**): comparison of implantation systems, (**E**–**H**): comparison of different rotation speeds, (**I**–**L**): comparison of different cooling conditions. Results were analyzed using the Kruskal-Wallis test with Dunn’s post hoc comparisons. Statistically significant differences are marked with asterisks. *—*p*-value < 0.05; **—*p*-value < 0.01; ***—*p*-value < 0.001. Parameter 1—Surface Smoothness, Parameter 1—Compressed Tissue Presence, Parameter 3—Carbonization, Parameter 4—Cracking and Adjacent Tissue Damage.

However, the most significant differences are observed for different cooling temperatures. No cooling gives the lowest score for all parameters evaluated, while cooling at 4 °C gives the highest score (see Figure 5).

### 3.5. Analysis of Optimal Combination of Drilling Parameters

**Figure 6:**
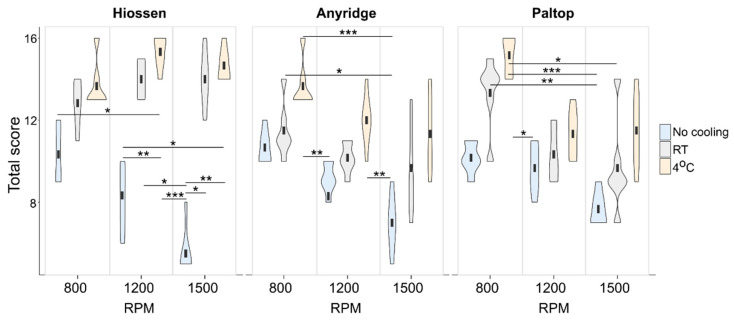
Violin plots showing the distribution of total score for different combinations of implantation system/rotation speed/cooling conditions. No cooling tends to give the lowest total score, regardless of the system used. The combination of high speed and no cooling gives the worst results. Results were compared within each implantation system using the Kruskal-Wallis test. Statistically significant differences between groups were evaluated using Dunn’s post hoc test and are marked with an asterisks. *—*p*-value < 0.05; **—*p*-value < 0.01; ***—*p*-value < 0.001.

Figure 6 shows how the combination of all drilling parameters influences the total score of the evaluated implantation quality traits. In general, no cooling tends to show the lowest score regardless of the system used—all but one of the statistically significant differences include the “no cooling” groups. The score for this group gets worse the higher the speed is; the results shown in Figure 5E–H favoring the lowest speed may be biased because of this dependance.

4 °C cooling tends to give the best overall score of all the implantation systems evaluated. It is also apparent that different implantation systems favor different rotation speeds. The Hiossen system gives better results at 1200 and 1500 rpm, whereas Anyridge and Paltop perform best at 800 rpm (see Table 2).

## 4. Discussion

This study aimed to compare the Hiossen, Anyridge, and Paltop implant systems in terms of surface smoothness, tissue compression, carbonization, and adjacent tissue damage to assess bone bed quality. Additionally, we systematically evaluate the effects of three cooling methods (room-temperature saline, 4 °C saline, and no cooling) on bone integrity—an aspect rarely explored in detail. Using fluorescence microscopy (DAPI and rhodamine staining), we precisely assess tissue compression, carbonization, and microfractures, providing insights often overlooked in prior research. To ensure a standardized and objective evaluation, we introduce a quantitative scoring system for bone bed quality. These findings enhance the understanding of drilling parameter effects on osseointegration and provide valuable clinical guidelines for optimizing implant success rates. Based on the statistical analysis, Hiossen drills achieved the best bone bed surface quality, with minimal loose tissue adhering to the implant bed walls, when operated at 1200 rpm with cooling at 4 °C. This drilling condition yielded the highest score among all examined samples (15.3). Similarly, the Paltop system demonstrated comparable results, scoring 15.2 points at 800 rpm under the same cooling conditions. The Anyridge system also produced favorable outcomes, though slightly lower than the others, achieving 13.7 points at 800 rpm with saline cooling at 4 °C.

The quality of bone bed preparation is integral to achieving successful osseointegration, which Brånemark [32] defines as the direct functional and morphological fusion of living bone with the surface of an implant. Osseointegration is both a chemical and biomechanical process, where biomechanical anastomosis involves bone tissue growing into the implant surface, while chemical anastomosis entails an immediate reaction between calcium, magnesium, and the implant material [27,32,33,34,35]. In the context of intramedullary implants, osteoblasts are of paramount importance and exert a decisive influence on the successful osteointegration of the implant, determining the tissue response to the implant surface [10,36,37]. Macrophages are another pivotal cell type in terms of bone remodeling and osteogenesis. As stated in research by Orvalho et al. [38], a deficiency or absence of these cells in bone tissue results in impaired and reduced bone regeneration. In the event of bone injury, undifferentiated M0 macrophages that are proximate to the site of injury will differentiate into the inflammatory M1 macrophages. These M1 macrophages will subsequently signal monocytes to the site of injury, thereby initiating the short-lived inflammatory response. Subsequent to this sequence of events, M1 macrophages will undergo differentiation into M2 macrophages, which are the primary cells responsible for the formation of osteoblasts [38]. The intact state of the cells following the bone bed preparation process exerts a significant influence on this response. The final quality of the bone-implant interface is not as significantly influenced by the accuracy of the bed preparation geometry or the types of implant surfaces as it is by the irreversible traumatization of the living tissue [14,39]. From a molecular perspective, the osteointegration process is accelerated in a tissue environment devoid of necrotic elements and destruction of implant-surrounding tissue. This phenomenon is reflected in assessed parameters of surface smoothness, compressed tissue presence, carbonization, cracking, and adjacent tissue damage. These elements can be mitigated by adhering to the established parameters and guidelines for a given system.

Moreover, the role of drill geometry on implant bed preparation quality needs to be highlighted. The geometry of the drill, including its cutting-edge sharpness, rake angle, and flute design, significantly influences the efficiency of bone cutting, heat generation, and irrigation effectiveness. Drills with sharper cutting edges and well-designed flutes facilitate smoother osteotomy formation, reducing mechanical trauma and improving chip evacuation [8,21]. The Paltop system utilizes step drills, designed to progressively guide the next drill, reducing vibration and chattering. This controlled cutting mechanism may explain why Paltop exhibited optimal results at lower speeds (800 rpm), where precision and reduced thermal effects are critical. The Hiossen system, on the other hand, features a multi-level tapered drill with high cutting power, allowing for a more aggressive drilling approach that skips intermediate steps. This design prevents bouncing and reduces heat accumulation, which may account for its superior performance at 1200 rpm [19,24]. These differences suggest that drill geometry plays a crucial role in determining which speeds optimize osteotomy quality and which conditions favor effective irrigation. While AnyRidge does not have a standardized drill design or fixed protocol, its performance variation across different conditions suggests that the adaptability of its drill selection could influence outcomes based on bone density and cooling strategies. Our findings emphasized that drill design should be considered alongside rotational speed and cooling conditions to achieve the best implant bed quality, minimizing tissue trauma and optimizing conditions for osseointegration.

A greater quantity of ground tissue remains adhered to the walls of the created hole, particularly when cooling was absent or inadequate. The Hiossen system exhibited tissue cracking near the surgical site in conditions where no cooling was applied, as well as at 800 rpm with room-temperature saline cooling and at 1500 rpm, where heat accumulation was more pronounced. The Anyridge system generally showed no damage to adjacent tissues; however, in nearly all cases, drill jerking effects were evident on the walls of the osteotomies, particularly at 1200 rpm, suggesting variations in cutting efficiency at different speeds. The Paltop system demonstrated optimal performance at 800 rpm, showing the greatest ability to preserve adjacent tissues. Nevertheless, when room-temperature saline was used, slight carbonization was observed on the surface of the osteotomies across all speeds, indicating that higher saline temperatures may reduce cooling efficiency. In contrast, cooling with chilled saline (4 °C) significantly improved osteotomy quality across all systems by enhancing heat dissipation and reducing the risk of thermal damage. At 1500 rpm, particularly in the Paltop system, the greatest surface damage was observed, likely due to increased frictional heat and suboptimal cooling, emphasizing the necessity of lower saline temperatures to alleviate these effects.

As posited by Coyac et al. [40] and Hoegel et al. [41], the residual bone chips in the bone bed do not inherently impede osteointegration. In fact, they may even facilitate bone formation, contingent on the viability of the cells within the chips [40,41]. The findings of Coyac et al. [40] indicate that residual bone chips following the preparation of a bone bed can directly enhance the formation of bone around an implant. In comparison to a conventionally prepared bed, where shavings are removed through rinsing and manually extracted from the recesses of the drill, the newly designed preparation tool demonstrated superior osteointegration outcomes, accompanied by accelerated bone remodeling processes. The novel drill design permitted the retention of chips within the preparation hole, which served as autogenous material [40]. As demonstrated in the study by Hoegel et al. [41], alkaline phosphatase, an enzyme indicative of osteoblast activity, is detected in chips extracted from the dissected bone bed. It is important to note, however, that this effect is not sustained when excessive bone preparation is employed [41]. This indicates that bone fragments generated following preparation can be categorized as living matter, and that their complete removal is unnecessary after preparation. Such fragments do not impede osteointegration and may even enhance it. It is of the utmost importance to prevent necrosis and damage to tissues in close proximity to the opening, thereby avoiding excessive friction and reducing preparation time. Among the results obtained, even in the most favorable cases, tissue masses were visible adjacent to the walls of the drilled holes. Therefore, as long as necrosis does not occur and there are viable cells in the ground tissues, a certain amount of tissue remaining in the hole will not worsen the situation. Consequently, one can hypothesize that it might even improve the osteointegration potential.

The limitation of the study lies in its reliance on swine rib specimens, which may not fully replicate the structural and biological properties of human bone, particularly regarding cellular composition and tissue response to mechanical and thermal stress. Additionally, while the study evaluated the effects of cooling protocols and drilling speeds, it did not account for other factors, such as operator technique and drill design, which could influence outcomes. Furthermore, the observed carbonization and tissue cracking in certain systems highlight the need for further optimization of preparation parameters to minimize tissue trauma, a critical factor in accelerating osseointegration. These limitations suggest the necessity for additional in vivo studies to validate the findings and improve the clinical applicability of bone bed preparation techniques.

## 5. Conclusions

The study demonstrated that the Paltop drill system achieved the most favorable bone bed quality at 1200 rpm with cooling at 4 °C, followed closely by the Hiossen system at 800 rpm under the same cooling conditions. The Anyridge system exhibited optimal performance at 1200 rpm with saline cooling at room temperature. These findings emphasize the critical role of optimizing drilling parameters in minimizing thermal damage, reducing tissue trauma, and preserving bone bed integrity—factors essential for successful osseointegration. Furthermore, the study highlighted the importance of adhering to evidence-based guidelines to minimize tissue necrosis and maintain viable bone chips, which may facilitate bone regeneration and accelerate osseointegration. However, certain limitations should be acknowledged. The use of swine rib specimens, while providing a valuable in vitro model, only partially replicates the structural and biomechanical properties of human bone. Additionally, the study did not account for operator variability or specific drill design differences, which could influence outcomes in clinical practice.

Future in vivo studies are recommended to validate these findings in human bone and further refine implant bed preparation techniques, ensuring improved long-term implant success and clinical outcomes.

## Figures and Tables

**Table 1:** Parameters related to the quality of implant bed preparation.

Parameter	Score	Description
Surface Smoothness (torn edges, debris)	1	Heavily ragged edges with attached bone and soft tissue fragments filling the borehole
	2	Ragged borehole edges with remnants of bone and soft tissue adjacent to the borehole edges
	3	Uneven borehole edges with isolated remnants of bone and soft tissue adjacent to the borehole edges
	4	Smooth surface with no noticeable remnants
Compressed Tissue Presence (compaction)	1	Distinct tissue compression causing deformation in bone lamellae and adjacent tissues, extending up to 3 lamellae around the borehole
	2	Distinct tissue compression causing deformation in bone lamellae adjacent to the borehole, extending up to 2 lamellae
	3	Slight compression of lamellae immediately adjacent to the borehole
	4	No tissue compression
Carbonization (thermal damage)	1	Carbonization within bone lamellae extending up to 3 lamellae around the borehole
	2	Carbonization within bone lamellae extending up to 2 lamellae around the borehole
	3	Carbonization within bone lamellae extending up to 1 lamella around the borehole
	4	No visible carbonization
Cracking & Adjacent Tissue Damage	1	Cracks observed within 3 bone plates outward from the borehole
	2	Cracks observed within 2 bone plates outward from the borehole
	3	Cracks observed within 1 bone plate outward from the borehole
	4	No cracks or damage to adjacent tissues

**Table 2:** Ranking of the best system/rotation speed/cooling combinations, sorted from highest to lowest total score. Numbers in table refers to mean score values.

System-Rotation Speed-Cooling	P1 Score	P2 Score	P3 Score	P4 Score	Total Score
Hiossen-1200-4 °C	3.83	3.83	3.83	3.83	15.3
Paltop-800-4 °C	3.67	3.83	3.83	3.83	15.2
Hiossen-1500-4 °C	3.83	3.67	3.67	3.5	14.7
Hiossen-1200-RT	3.5	3.83	3.67	3	14
Hiossen-1500-RT	3.33	3.33	3.5	3.83	14
Anyridge-800-4 °C	3.33	3.33	3.67	3.33	13.7
Hiossen-800-4 °C	3.33	3.33	3.67	3.33	13.7
Paltop-800-RT	3.5	3.17	3.5	3.17	13.3
Hiossen-800-RT	3.5	3.5	3	2.83	12.8
Anyridge-1200-4 °C	2.83	3	3.17	3	12
Anyridge-800-RT	3	2.67	3	2.83	11.5
Paltop-1500-4 °C	2	3.33	3	3.17	11.5
Anyridge-1500-4 °C	2	3.33	3.17	2.83	11.3
Paltop-1200-4 °C	2.67	2.83	3	2.83	11.3
Anyridge-800-No cooling	2.67	2.5	2.67	2.83	10.7
Hiossen-800-No cooling	2.5	2.67	2.5	2.67	10.3
Paltop-1200-RT	2.67	2.83	2.33	2.5	10.3
Anyridge-1200-RT	2.67	2.5	2.5	2.5	10.2
Paltop-800-No cooling	2.5	2.33	2.5	2.83	10.2
Anyridge-1500-RT	2.5	2.33	2.67	2.17	9.67
Paltop-1200-No cooling	2	2.5	2.5	2.67	9.67
Paltop-1500-RT	2.5	2.33	2.5	2.33	9.67
Anyridge-1200-No cooling	2	2.5	2.17	2.5	9.17
Hiossen-1200-No cooling	2.33	2.17	2.17	1.67	8.33
Paltop-1500-No cooling	2	2	1.67	2	7.67
Anyridge-1500-No cooling	2	1.5	1.33	2.17	7
Hiossen-1500-No cooling	1.5	1.33	1.17	1.5	5.5

## Data Availability

Data are contained within the article.
